# Aortic valve Libman-Sacks endocarditis with aortic root and coronary ostium involvement – A case report

**DOI:** 10.3389/fcvm.2026.1770621

**Published:** 2026-08-28

**Authors:** Michele D’Alonzo, Eric Bergoend, Cyrus Moini, Antonio Fiore

**Affiliations:** 1 Department of Cardiac and Thoracic Surgery, Henri Mondor University Hospital, Assistance Publique-Hôpitaux de Paris, Créteil, France; 2 Department of Cardiac Surgery, Poliambulanza Foundation Hospital, Brescia, Italy; 3 Department of Cardiology, Hospitalier Sud Ile de France, Melun, France; 4 Université Paris Est, Inserm IMRB U955, CEpiA Team, Créteil, France

**Keywords:** antiphospholipid syndrome, aortic valve regurgitation, coronary embolism, Libman-Sacks endocarditis, systemic lupus erythematosus

## Abstract

**Background:**

Libman-Sacks (LS) endocarditis, a nonbacterial thrombotic endocarditis, is the most frequent cardiac manifestation of systemic lupus erythematosus (SLE), particularly when associated with antiphospholipid syndrome (APS). It usually involves the mitral valve and is often clinically silent.

**Case summary:**

We present a 25-year-old woman with SLE and APS who developed acute hemodynamic deterioration. Urgent surgery revealed a large endocarditic mass arising from the aortic valve leaflet, extending to the aortic root and obstructing the right coronary ostium. Resection and valve replacement were performed successfully.

**Discussion:**

Unlike typical LS endocarditis, which manifests as mild valvular lesions, this case demonstrates rare aortic root involvement with coronary ostial obstruction. A review of the literature confirms that such an extension has not been previously reported, underscoring the heterogeneous and potentially life-threatening nature of LS endocarditis.

**Take home messages:**

LS endocarditis can present atypically. Recognition of unusual patterns is critical for timely intervention.

## Introduction

In 1924, Libman and Sacks originally reported four systemic lupus erythematosus (SLE) cases with verrucose vegetative endocarditis (1). After 100 years since its discovery, much has been unveiled about this pathology, although numerous uncertainties still persist. LS endocarditis, characterized by verrucose vegetative lesions, primarily affecting the mitral valve, poses challenges in diagnosis and management. The incidence of LS endocarditis is notably higher in SLE patients with antiphospholipid syndrome (APS) (2), highlighting the complex interplay of autoimmune processes. Diagnosis often requires a high index of clinical suspicion, as LS endocarditis may remain asymptomatic or present with extra-cardiac manifestations.

The optimal management strategy remains a subject of ongoing debate, with immunomodulatory therapy aimed at controlling inflammation and thromboembolic risk, while surgical intervention is reserved for significant valvular defects. This case report highlights a unique scenario of a 25-year-old woman with SLE and APS also presenting with LS endocarditis growing from the aortic valve leaflet until the aortic root, leading to mechanical obstruction of the right coronary ostium.

## Case presentation

**Figure 1:**
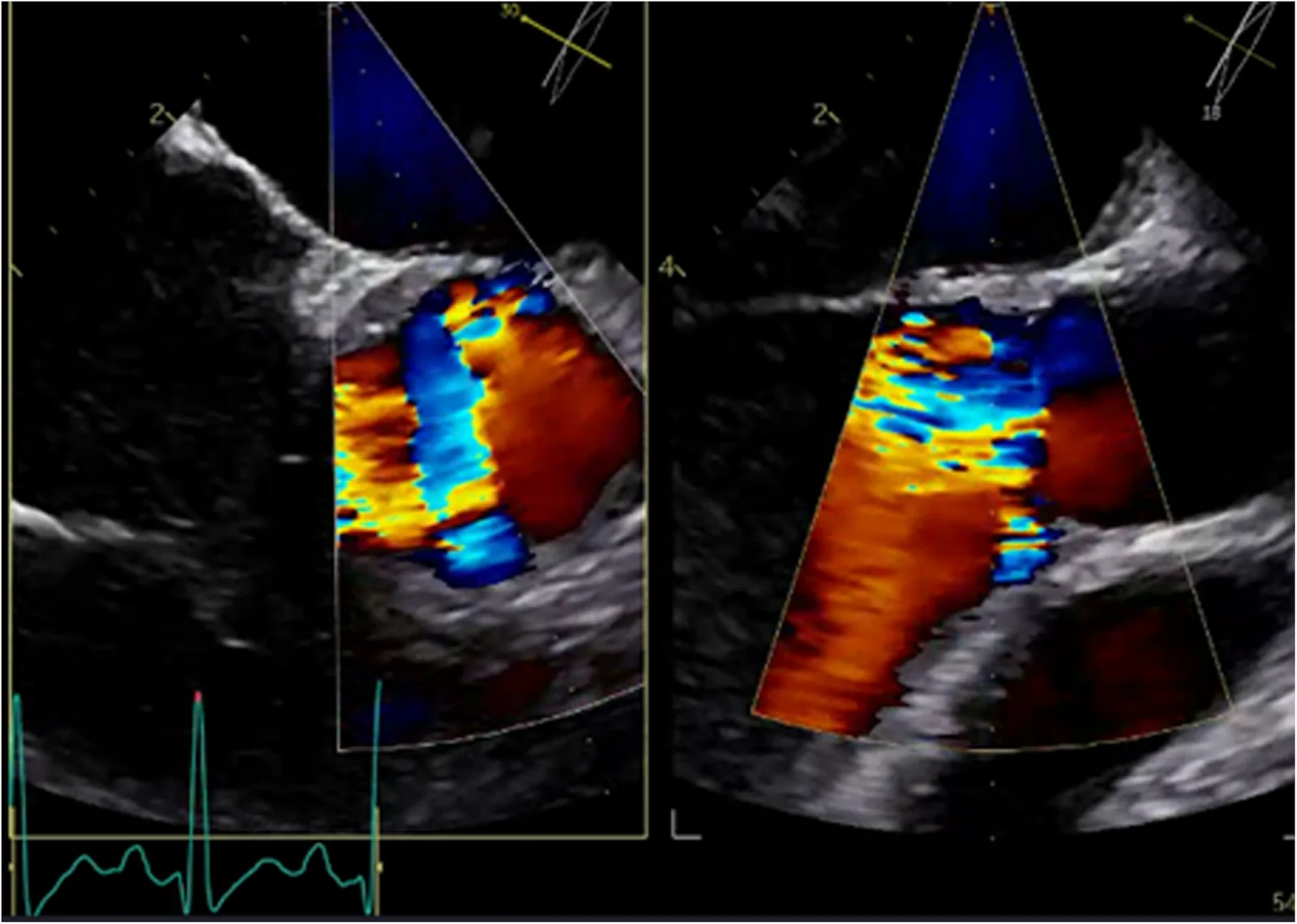
Preoperative echocardiographic image, that show a severe aortic regurgitation, with thickened valve's leaflets.

We report the case of a 25-year-old female with an 8-year history of SLE complicated by APS. Her immunological profile revealed positive lupus anticoagulant, negative anticardiolipin antibodies, and positive anti-*β*2-glycoprotein I antibodies, findings documented during prior specialist evaluations. The patient was managed with anticoagulants and immunosuppressors, and experienced complications such as cutaneous-articular lesions, impaired kidney function, splenic infarction, and anaemia. Two years prior, during a cardiologic check-up, a transthoracic echocardiogram revealed severe aortic regurgitation, characterized by left coronary cusp prolapse (Figure 1, Supplementary Videos S1, S2). The echocardiographic parameters were consistent with severe AR, with an EROA of 0.35 cm^2^, vena contracta 6 mm, and a left ventricle at the upper limits of normal (LV diastolic diameter 6.1 cm, LV diastolic volume 130 mL).The patient was symptomatic for dyspnoea and an indication for cardiac surgery was already existent, but the patient declined any intervention at that time.

The patient's condition deteriorated over the following months, culminating in acute pulmonary edema and low-flow syndrome. She did not receive any inotropes, as she was taken to the operating room within a few hours of presenting to the emergency department. On presentation, she exhibited severe dyspnea, tachypnea (32 breaths/min), hypoxemia (SpO₂ 88% on room air), tachycardia (115 bpm), and elevated blood pressure (160/95 mmHg). At the hospital admission, the electrocardiogram did not show any specific abnormalities, whereas transthoracic echocardiography revealed a reduced left ventricular ejection fraction of 25%, associated to an image of large endocarditis on the right coronary aortic leaflet, accompanied by a moderate elevation in Troponin Hs (200 ng/L). Preoperative laboratory tests showed no striking abnormalities and were consistent with acute heart failure (white blood cell count 13,000/µL, C-reactive protein 50 mg/L, procalcitonin 1.2 ng/mL).

**Figure 2:**
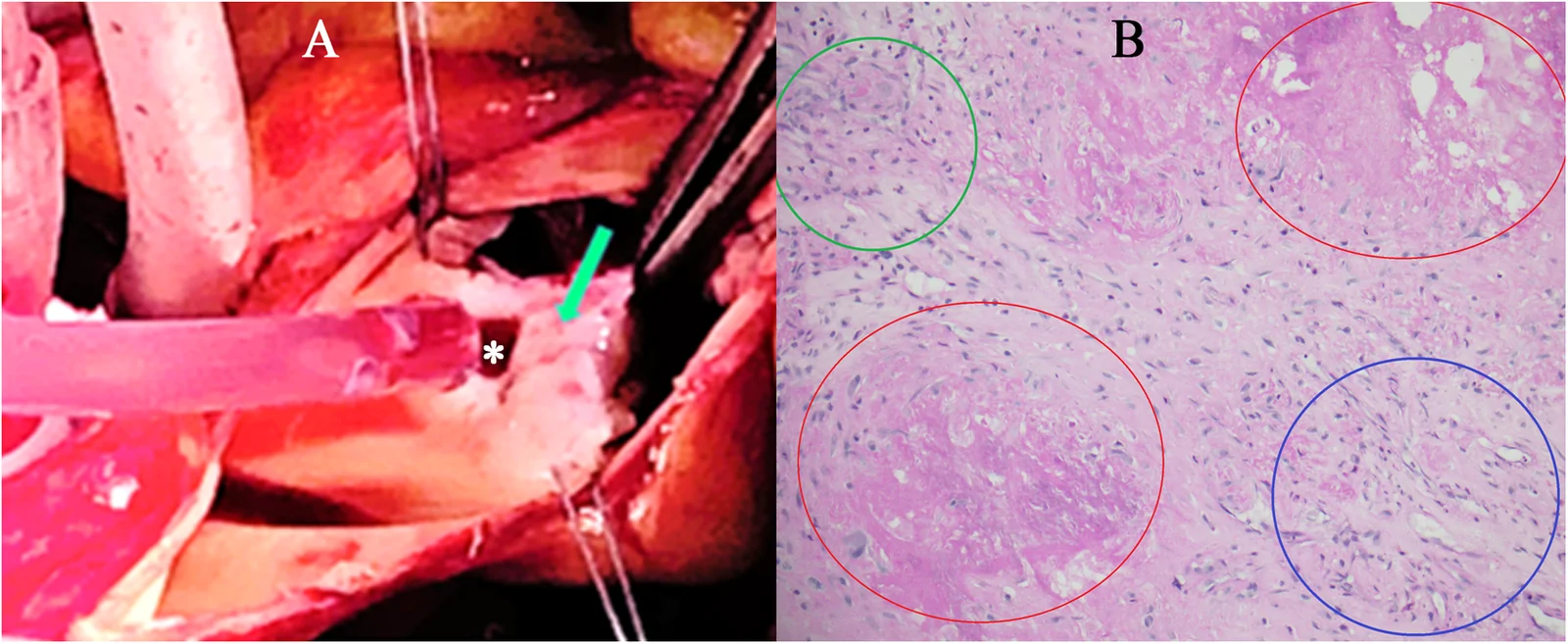
**(A)**: intraoperative image of the fibrous mass (green arrow) cleaved from the sinus of valsalva at the level of right coronary ostium (*). **(B):** Microscopic histopathological examination: haematoxylin and eosin stain of the aortic surface of the excised aortic valve leaflet. Valvular LS lesions typically feature fibrin deposits undergoing fibroblastic organization, neovascularization, sporadic hematoxylin bodies, and varying degrees of mononuclear cell infiltration. Specifically, the depicted image reveals widespread fibrinoid necrosis, characterized by amorphous eosinophilic material (red circle), a prominent chronic inflammatory infiltrate rich in lymphocytes and plasma cells (green circle), and areas of fibrous tissue indicative of chronic repair and remodeling (blue circle).

The patient underwent emergency surgery, intraoperatively an uncommon endocarditic mass was discovered, originating from the aortic valve leaflet, that appeared retracted and thickened. Moreover, this greyish-white structure has grown to wall covering the aortic root and even obstructing the coronary ostium (Figure 2A). Fortunately, the presence of a distinct cleavage plane allowed for the preservation of the aortic root and coronary ostium. Therefore, the surgery consisted in the excision of the mass associated with aortic valve replaced by bioprostheses, considering the patient's desire for future pregnancy. The patency of the coronary artery was carefully assessed manually using a surgical instrument known as a Klemmer clamp, which was gently applied to test the ostium of the involved coronary artery; therefore, it was deemed unnecessary to proceed with coronary artery bypass grafting.

The histology analysis showed the seat of chronic fibrous remodelling, focal calcified, with many thick-walled neo-vessels. Those findings consistent with Libman-Sacks Endocarditis (Figure 2B). The post-operative course was uneventful, and the patient was discharged after 8 days without the need for antibiotic therapy. The one-year follow-up echocardiogram showed normal aortic valve function, normal ejection fraction and no recurrence of endocarditis.

## Discussion

The association between Libman-Sacks (LS) endocarditis and systemic lupus erythematosus (SLE) involves complex considerations. LS endocarditis, a non-bacterial thrombotic endocarditis, is the most notable cardiac manifestation of SLE.

Libman–Sacks endocarditis (LS) falls within the broader spectrum of conditions known as nonbacterial thrombotic endocarditis (NBTE). Approximately 46% of NBTE cases are associated with malignancies, particularly mucinous adenocarcinomas. In the remaining cases, NBTE is linked to autoimmune diseases such as SLE or APS or both, as well as vasculitis and rheumatoid arthritis. Notably, COVID-19 infection has also been reported in association with this condition. Interestingly, concomitant infective endocarditis is observed in only about 1% of NBTE cases (3).

In recent research, it was found that LS endocarditis was identified in 1.1% of young women with SLE (969 woman with median age of 28.4 years old). Follow-up echocardiography revealed persistent vegetations (notoriously aseptic fibrin deposits on cardiac valves resulting from hypercoagulability and endocardial damage) in some patients, while valve function deteriorated in others. Incidence of NBTE is poorly known, estimated at 1.6% in old autopsy studies and reaching 9% in other studies (4).

At present, NBTE and LS lack established diagnostic criteria, dedicated guidelines and therapeutic pathways. Surgical intervention was not required in all cases, suggesting the need for careful consideration in the management of those forms of endocarditis (5).

Corticosteroid therapy, commonly used to enhance overall survival in SLE patients, may paradoxically contribute to the formation of scar tissue on heart valves, also increase tissue fibrosis and organic damage, finally worsening valvular damage and dysfunction. Nonetheless, appropriate and sufficient steroid therapy to control autoimmune disease activity is demanded (6).

Antiphospholipid antibodies, implicated in APS, activate various cellular components, such as monocytes, platelets and endothelial cells, leading to clot formation and thromboembolic events. Heart valve disease, including LS endocarditis, affecting approximately one-third of those patients.

LS endocarditis is characterized by a non-infectious verrucous vegetation that can be distinguished pathologically from rheumatic heart disease and bacterial endocardial disease. Unlike infective endocarditis where usually the valve needs to be completely excised to remove infected tissue, mitral valve repair for LS endocarditis is quite always possible, thus eliminating the need for lifelong anticoagulation therapy. Nonetheless, on LS endocarditis population, the outcomes of surgical valvular replacement vs. mitral repair have been limited to case reports or series (7).

Extra-cardiac manifestations of APS are both venous and arterial thromboses, recurrent pregnancy loss, and even thrombocytopenia (8).

The incidence of thromboembolic cerebrovascular events in patients with LSE has been reported as 20%–35%. Beyond stroke, the most frequent clinical presentations include chest pain (29%) and acute heart failure (10%), whereas fever is observed in fewer than 17% of cases, in contrast to infective endocarditis, in which its prevalence is substantially higher (3). Roldan et al. presented the first fully integrated study linking Libman–Sacks vegetations with cerebrovascular events and also death (9).

While surgical intervention has been suggested in complicated cases, the indications for surgery and long-term prognosis remain areas of ongoing debate. Also, the surgical strategy is a major concern: in our case, this repair option was not feasible because the endocarditic mass had destroyed a leaflet of the aortic valve, which was already compromised due to prolapse of another cusp. Consequently, we proceeded with a valve replacement using a bioprosthesis, also considering the patient's desire for future pregnancy.

In summary, Libman-Sacks endocarditis presents a multifaceted challenge in the context of systemic lupus erythematosus and antiphospholipid syndrome. The interplay of clinical, diagnostic, and therapeutic aspects underscores the importance of a comprehensive and multidisciplinary approach in managing this complex condition.

## Data Availability

The original contributions presented in the study are included in the article/Supplementary Material, further inquiries can be directed to the corresponding author.

## Supplementary material

Supplementary Video 1 Pre-operative two-dimensional transthoracic echocardiographic view demonstrating prolapse of the left coronary cusp and vegetations consistent with Libman-Sacks endocarditis.

Supplementary Video 2 Pre-operative color Doppler transthoracic echocardiographic view showing severe aortic regurgitation and turbulent flow across the prolapsed left coronary cusp.
